# Safe spaces for beneficiaries of a combination HIV prevention intervention for adolescent girls and young women in South Africa: access, feasibility, and acceptability

**DOI:** 10.1186/s12889-022-13445-w

**Published:** 2022-05-21

**Authors:** Catherine Mathews, Zoe Duby, Brittany Bunce, Nathanael van Blydenstein, Kate Bergh, Anthony Ambrose, Fiona Mpungu, Kim Jonas

**Affiliations:** 1 Health Systems Research Unit, South African Medical Research Council, Cape Town, South Africa; 2 School of Public Health and Family Medicine, University of Cape Town, Cape Town, South Africa; 3 Sheffield Institute for International Development, University of Sheffield, Sheffield, UK; 4 Networking HIV and AIDS Community of Southern Africa (NACOSA), Cape Town, South Africa; 5 Department of Psychology, University of Cape Town, Cape Town, South Africa; 6 Division of Child and Adolescent Psychiatry, University of Cape Town, Cape Town, South Africa

**Keywords:** HIV prevention, Safe space, Feasibility, Accessibility, Adolescent girls, Young women

## Abstract

**Background:**

Safe Spaces are a feature of combination HIV prevention interventions for adolescent girls and young women (AGYW) in South Africa. We investigated whether AGYW at risk for adverse sexual and reproductive health (SRH) outcomes accessed Safe Spaces that were part of an intervention, as well as their feasibility and acceptability.

**Methods:**

In December 2020 to February 2021, as part of a process evaluation of a combination HIV prevention intervention, we randomly sampled 2160 AGYW intervention beneficiaries aged 15–24 years from 6 of the 12 intervention districts. We invited them to participate in a phone survey, with questions about their vulnerability to adverse SRH outcomes, and participation in intervention components including Safe Spaces. We examined factors associated with use of Safe Spaces using bivariate analyses and Pearson’s chi squared tests. We also conducted in-depth interviews with 50 AGYW beneficiaries, 27 intervention implementers, 4 health workers, 7 social workers, and 12 community stakeholders, to explore perceptions and experiences of the intervention. Thematic analysis of the qualitative data was performed.

**Results:**

At least 30 Safe Spaces were established across 6 districts. Five hundred fifteen of two thousand one hundred sixty sampled AGYW participated in the survey of whom 22.6% visited a Safe Space, accessing HIV testing (52.2%), mobile health services (21.2%) and counselling for distress (24.8%) while there. Beneficiaries of lower socioeconomic status (SES) were less likely to have visited a Safe Space, compared with those of higher SES (13.6% versus 25.3%; *p* < 0.01). Implementers described political, structural and financial challenges in identifying and setting up Safe Spaces that were safe, accessible and adequately-resourced, and challenges with AGYW not utilising them as expected. AGYW shared positive views of Safe Spaces, describing benefits such as access to computers and the internet, support with homework and job and education applications, and a space in which to connect with peers.

**Conclusion:**

AGYW are attracted to Safe Spaces by educational and employment promoting interventions and recreational activities, and many will take up the offer of SRH services while there. The poorest AGYW are more likely to be excluded, therefore, an understanding of the obstacles to, and enablers of their inclusion should inform Safe Space intervention design.

## Introduction

Adolescents and young people in sub-Saharan Africa often experience services provided in health facilities as unfriendly and unresponsive to their needs, and this is especially the case for sexual and reproductive health (SRH) care, where they have substantial unmet needs [1]. Marginalized youth in particular are at higher risk of SRH problems and experience greater obstacles in accessing SRH interventions [1]. Young people need spaces that are “inclusive environments” where they feel accepted, free from stigma, maltreatment and violence, in which they can access youth-friendly health services; Safe Spaces have been promoted to provide these environments and to promote equity in access to health services for those who need them most [2].

A Safe Space refers to “a formal or informal place where women and girls feel physically and emotionally safe”; ‘Safe’ refers to the “absence of trauma, excessive stress, violence (or fear of violence), or abuse” [3]. Safe Spaces have been promoted as places where the needs of adolescent girls and young women (AGYW) in particular can be met, especially those who are more vulnerable, who are at risk of violence, and who might be excluded from other youth programming because they are not in school or not living with their parents [4]. Programmes that include Safe Spaces for AGYW have aimed to provide a space for AGYW to socialize, receive social support, foster supportive social networks, acquire skills, access safe and non-stigmatizing services such as for violence against women, SRH, legal and psychological services, and receive information on women’s rights, health and services [3–5].

In recent years, Safe Spaces have become a common feature of women-centred HIV, SRH and trauma-sensitive care service models globally, regarded as an appropriate way to overcome barriers that limit women’s access to care [6]. Safe Spaces are not always referred to as such, however the concept of safe physical and social space has been conceptualized as a key delivery mechanism in HIV programmes in East and Southern Africa that focus on social protection interventions for young women vulnerable to HIV [7]. In sub-Saharan Africa, hundreds of Safe Spaces have been set up as a structure to deliver interventions and services to AGYW as part of the USAID and PEPFAR-funded DREAMS (Determined, Resilient, Empowered, AIDS-free, Mentored and Safe) program [8], as a part of AGYW programmes funded by the Global Fund [9], and by other programmes [10]. They have been set up to address sexual violence, gender equality, limited economic opportunities [5], and to provide PrEP services [11].

Youth centres, “meeting points that offer a youth-friendly, safe, non-threatening environment for information and service delivery across various sectors such as health, education, job training, or recreation” [12], are conceptually similar to Safe Spaces. However, evidence is mixed on whether or not youth centres are effective in reaching adolescents, particularly females, with SRH programmes and services [12–14]. The mixed evidence highlights the importance of understanding context-specific factors to inform design of interventions in South Africa.

We do not have sufficient evidence about whether Safe Spaces designed exclusively for AGYW have the potential to provide accessible SRH services for AGYW who most need such services. It is important to know the extent to which vulnerable or marginalized AGYW have access to them, such as those who are poor, orphaned, or not in education, employment, or training. AGYW who have an early sexual debut, or who are at risk of pregnancy, HIV or STIs, or who are at risk of intimate partner violence, also need equitable access to spaces that provide SRH education and services. AGYW with mental health problems, or who are defined as languishing in terms of their well-being, are more vulnerable to adverse SRH outcomes, and are less likely to access SRH care [15].

The study of intervention feasibility involves determining whether an intervention, such as Safe Spaces, is appropriate for wider implementation [16] and includes a focus on acceptability [17, 18] and appropriateness, referring to the relevance of the intervention for the context, providers and target beneficiaries, as well as the intervention’s suitability to address the specific problem [17]. Feasibility studies provide information about whether an intervention can be fully implemented as planned, including when resources, time, and commitment are constrained [16]. They can provide information about perceived appropriateness, success or failure of execution, factors affecting implementation ease, difficulty or quality, and the resources needed to implement [16].

We investigated whether AGYW beneficiaries of a South African combination HIV prevention programme who were most at risk for adverse SRH outcomes, had access to the Safe Spaces. We also explored factors that attract AGYW to Safe Spaces, the acceptability of these spaces to AGYW and implementers, and the feasibility of implementing a Safe Space intervention.

### Setting

Combination HIV prevention interventions, which merge effective biomedical, behavioural and structural interventions for combined delivery, are one of the key strategies for reaching the 90–90-90 targets and achieving the Sustainable Development Goal (SDG) of ending the HIV epidemic by 2030 [19]. The Global Fund to Fight AIDS, TB and Malaria invested in a combination HIV prevention intervention for South African AGYW aged 15 to 24 years, implemented 2019–2022, in 12 South African districts with highest HIV prevalence amongst AGYW. The AGYW programme aims include decreasing HIV incidence, teenage pregnancy, and gender-based violence and increasing retention in school and economic opportunities. AGYW were introduced to the intervention through a number of entry points, recruited through various demand creation activities such as career jamborees, community dialogues, outreach activities at shopping centres, door-to-door home visits and during community events, however many recruitment events were cancelled because of the COVID-19 pandemic and lockdowns.^1^

Dedicated Safe Spaces in communities were an important feature of the intervention approach, providing a space for SRH services to be delivered. Services were also delivered in schools, colleges, and mobile clinics, and some were delivered by external service providers such as government health service providers, in their own settings via referrals from implementers. The Safe Space programme component was conceptualized with a ‘hub-and-spokes model’ to conduct HIV prevention interventions and services to out-of-school AGYW. Implementers were mandated to establish a Safe Space “hub” in each district from where their services could be provided, and to offer their programmes to AGYW who were geographically further away using satellite Safe Spaces (4 per district). The central hub was intended to be a permanent space with satellites/mobile services providing outreach services. Ideally, the selected centres would be close to a high-volume health facility. These spaces would be staffed by professional staff such as social workers, social auxiliary workers and nurses, as well as trained peer group trainers. The satellite Safe Spaces were smaller spaces where a limited number of services could be offered a few days per week.

## Methods

We conducted a mixed-methods study comprising quantitative and qualitative methods. A survey was used to collect quantitative data. Qualitative data was gathered through in-depth interviews. Sampling for both the survey and qualitative components followed the same approach. We provided the implementers with the list of sampled AGYW beneficiaries’ unique study numbers. Then the implementers called each beneficiary, provided brief details about the study using a script provided by the research team. The implementers asked whether the beneficiary would be willing to be contacted by a study team member to be invited to participate in the telephone survey or an interview. If the beneficiary was under 18 years of age, we first obtained parental consent telephonically before we conducted the consent process with the beneficiary.

### Quantitative sub-study

Between 1 December 2020 and 28 February 2021, we conducted a cross-sectional, descriptive telephone survey among AGYW programme beneficiaries in six of the 12 intervention districts. The sampling frame comprised an anonymized version of the AGYW programme database of all beneficiaries (127,951 beneficiaries) with contact details for each beneficiary. We stratified beneficiaries by district and age group, and for the younger age group, by whether they were in school (Table 1). We sampled double the number of AGYW in the younger age group because we expected that approximately 50% of them would never have had sex, and therefore would not contribute to our measures of effective coverage of PrEP and contraception, which were the main outcomes of the evaluation. We randomly sampled 2160 AGYW programme beneficiaries (360 per district) from all beneficiaries who had been enrolled in the programme for at least 1 year (to ensure they had had time to participate in the programme activities), stratified by age group and school status. This implies they were enrolled predominantly during the first year of the 2019–2022 grant period, or in the early part of the second year. Data was anonymized during sampling.

**Table 1:** Sample realization for each of the sampling strata in the survey of AGYW programme beneficiaries

District	A Bojanala	B Klipfontein	C King Cetshwayo	D Ehlanzeni	E Nelson Mandela Bay	F Thabo Mofutsanyana	Total
	Freq (%)	Freq (%)	Freq (%)	Freq (%)	Freq (%)	Freq (%)	
Principal Recipient	PR 1	PR 1	PR 2	PR 2	PR 3	PR 3	
AGYW 15–19 years in school	24/200 (12.0%)	6/200 (3.0%)	43/200 (21.5%)	79/200 (39.5%)	30/200 (15.0%)	30/200 (15.0%)	**212/1200 (17.7%)**
AGYW 15–19 years out of school	9/40 (22.5%)	9/40 (22.5%)	15/40 (37.5%)	1/40 (2.5%)	5/40 (12.5%)	13/40 (32.5%)	**52/240 (21.7%)**
AGYW 20–24 years	30/120 (25.0%)	43/120 (35.8%)	68/120 (56.7%)	28/120 (23.3%)	35/120 (29.2%)	47/120 (39.2%)	**251/720 (34.9%)**
**Total AGYW**	**63/360 (17.5%)**	**58/360 (16.1%)**	**126/360 (35.0%)**	**108/360 (30.0%)**	**70/360 (19.4%)**	**90/360 (25.0%)**	**515/2160 (23.8%)**

### Survey measures

The questionnaire included items measuring demographic characteristics, sexual and reproductive health and risk, measures of intervention coverage, the AGYW’s experience of, and feelings about the interventions, and factors that were facilitators or barriers to uptake of interventions. It was available in the language of the participant’s choice including isiZulu, isiXhosa, Afrikaans, Setswana, SeSotho, siSwati and English. To measure knowledge about and access to Safe Spaces, we asked “Do you know if an organisation in your community provides a safe space for young women like you to hang out and receive support?” and “In the past year, have you spent time at a safe space in your community?” If they had spent time at a safe space, we asked whether they had participated in a range of activities and received various services at the safe space.

We chose 13 variables to derive our socio-economic status (SES) indicator, several of which are commonly used in other surveys to create similar indices: 1. AGYW was away from home for more than 1 month in past 12 months (internal migration has been shown to cause and be caused by poverty [20]), 2. Has piped water in household, 3. Has flushing toilet in household, 4. Household has working electricity, 5. Household has a car, 6. Household has a computer, 7. Household has the internet, 8. Household has a refrigerator, 9. Household has a stove, 10. AGYW or member of her household went a day/night without eating in the past month, 11. AGYW has own money, 12. AGYW saves money, and 13. AGYW owes money.

We included a social well-being measure (Mental Health Continuum Short-Form), as this measure has shown good psychometric properties for a South African context [21] and it aligns with young people’s perceptions and experiences of well-being in South Africa [22]. The scale contains questions about three dimensions of well-being: hedonic emotional well-being (being happy, interested in life, and satisfied with life); eudaimonic social well-being (social contribution, social integration, social actualization or growth, social acceptance, and social coherence); and eudaimonic psychological well-being (self-acceptance, environmental master, positive relations with others, personal growth, autonomy, purpose in life). The response options include “never”, “once or twice”, “about once a week”, “about 2 or 3 times a week”, “almost every day” and “every day”. To classify participants as “languishing”, they needed to report that they ‘never’ or ‘once or twice’ experienced at least seven of the symptoms, with at least one from the hedonic cluster.

We used the Center for Epidemiological Studies Depression Scale (CES-D-10) as our mental health measure. It is a brief depressive symptom screener which has been validated in South Africa [23]. It measures depressive symptoms in the past week. Questions include three items on depressed affect, five items on somatic symptoms, and two on positive affect, with scoring ranging from *“rarely or none of the time”* (score of 0) to *“all of the time”* (score of 3). Scoring is reversed for items reflecting positive affect statements. Total scores can range from 0 to 30 and higher scores reflect greater severity of depressive symptoms. We used a cut-off score of 12 to classify AGYW as having a high risk of depression, as recommended for South African populations by Baron and colleagues [23].

To measure alcohol use, we used a brief version of the twelve-item Alcohol Use Disorders Identification Test (AUDIT), namely AUDIT-C [24], to describe the prevalence of hazardous drinking among AGYW. A participant’s AUDIT-C score can range from 0 to 12. Informed by the recommendation emanating from the South African study [24], a cut-off score of greater than or equal to 2 indicates hazardous drinking.

### Analysis of survey data

We explored the relationship between indicators of vulnerability or risk and access to Safe Spaces by calculating proportions for each factor by Safe Space access, and performing Pearson’s chi-squared tests to compare proportions. Stata (Stata 15.1, StataCorp, Texas, USA) was used to perform the analyses [25]. A participant’s socioeconomic (SES) group was determined using Cluster Analysis with the K-Modes algorithm [26], with the 13 SES questions that were included in the AGYW survey. Cluster Analysis is an exploratory and unsupervised machine learning technique that allows analysts to divide data into meaningful groups based upon shared features. The package “klaR” was used for the Cluster Analysis of [27].

### Qualitative sub-study

Data collection for the qualitative study component occurred in the same period as the telephone survey, and explored perceptions and experiences of the Safe Space intervention. In-depth interviews (IDIs) were conducted with 100 participants from the same six districts as the survey sample, comprising fifty (50) AGYW between the ages of 15 and 24 years (sampled and contacted using the same approach as for the survey), 27 intervention Implementers, 4 health workers, 7 social workers, and 12 other community stakeholders. Semi-structured interview guides framed discussions, outlining key topics for discussion. Interviews were conducted in participants’ language of choice (isiZulu, isiXhosa, Afrikaans, Setswana, SeSotho, siSwati, English) by female interviewers fluent in the site languages, who had received training on research ethics, the study protocol, and qualitative interviewing skills. Given the context of the COVID-19 pandemic, IDIs were conducted telephonically, and audio-recorded with participants’ consent. Audio recordings were directly translated from their original language into English transcripts, which were reviewed for accuracy. Three analysts coded the qualitative data, following a cyclical process of iterative thematic analysis. A set of pre-determined deductive code types were reflexively refined to reflect emerging topics during preliminary analysis; analysis included in this paper focused on codes relating to Safe Spaces, implementation experiences, AGYW beneficiary experiences, and implementation context. Through collaborative interpretation, the analysts engaged in data immersion, re-examining data at different stages in the process, documenting reflective thoughts and sharing growing insights during regular discussions. The use of analytic memos created an important extra level of narrative, providing an interface between participants’ data, researchers’ interpretations, and wider theory.

### Ethical considerations

The SAMRC Research Ethics Committee granted research ethics approval to conduct this study (EC036–9/2020).

## Results

### Survey sample realization

The sample realization was 23.8%; the proportion of the sampled beneficiaries who were uncontactable varied by district from 32.7 to 74.6% (Table 1).

### Characteristics of survey participants and risk profiles by safe space access

Approximately half of the participants were in the younger age group (15 to 19 years). Most participants (71.5%) were in the relatively high SES group, over a third were maternal and/or paternal orphans, 12.0% were classified as not in education, employment or training, 30.9% had ever been pregnant, 5.1% had ever been in a transactional relationship with a boy/man or had had transactional sex, 15.0% reported they had been afraid of a male partner in the past 6 months, 14.4% were classified as languishing, 28.2% were classified at high risk of depression and 48.2% had an audit C score of 2 or more (Table 2). Most (75.5%) had ever had sex, and of these 6.7% had had an early sexual debut, and during the 6 months before the survey 14.9% reported a male partner 5 or more years older than them, 20.8% reported more than one male sexual partner, 77.9% used condom inconsistently, and 66.3% used contraception inconsistently (Table 2). Only 144 beneficiaries (28.0%) knew of an NGO in their community that provided a “safe space for young women to hang out and receive support”. Nearly a quarter of participants (113; 22.6%) reported spending time at a Safe Space with no statistically significant difference between age groups (Table 2). There were significant differences between districts: 14.3, 26.3, 35.5, 25.2, 10.3 and 13.6% of beneficiaries in districts A to F respectively reported spending time at a Safe Space (*p* < 0.01). Beneficiaries who were classified as in the relatively lower SES category were significantly less likely to have accessed and spent time at a Safe Space, compared with those in the relatively high SES category (Table 2). Participants who reported multiple male partners in the 6 months before the survey were significantly more likely to have spent time at a Safe Space compared with those who had one or no sexual partners (Table 2). When considering all other indicators of vulnerability to adverse SRH outcomes, there were no differences in accessing Safe Spaces between AGYW classified as vulnerable according to an indicator and those not classified as vulnerable.

**Table 2:** Characteristics and risk profiles of 515 AGYW programme beneficiaries who accessed a Safe Space (unweighted analysis)

Variable	Total sample N (%)	Accessed a Safe Space = 1 Freq (%)	Pearson Chi2 comparing those who did and did not access a Safe Space for each variable (Test statistic) *P* value
Age group			
15–19 years	264 (51.3)	52 (18.7)	(1.5837) 0.21
20–24 years	251 (48.7)	61 (24.3)	
SES^*^			
Relatively low	147 (28.5)	29 (13.6)	**(8.3471) < 0.01**
Relatively high	368 (71.5)	93 (25.3)	
Hunger in household in past month			
Yes	86 (16.7)	23 (26.7)	(1.3902) 0.24
No	429 (83.3)	90 (21.9)	
Orphan status			
Maternal and/or paternal orphan	196 (38.1)	40 (20.4%)	(0.4345) 0.51
Not orphaned	319 (61.9)	73 (22.9%)	
Maternal orphan status			
Maternal orphan	102 (19.8)	24 (23.5)	(0.1872) 0.68
Not a maternal orphan	413 (80.2)	89 (21.6)	
NEET^a^			
Yes	62 (12.0)	18 (29.0)	(2.0690) 0.15
No	453 (88.0)	95 (21.0)	
Ever been pregnant			
Yes	159 (30.9)	32 (20.1)	(0.4429) 0.51
No	356 (69.1)	81 (22.8)	
Ever had a transactional relationship with a man or transactional sex			
Yes	26 (5.1)	8 (30.8)	(1.2458) 0.26
No	489 (95.0)	105 (21.5)	
Fear of male partner in past 6 months			
Yes	77 (15.0)	21 (27.3)	(1.5023) 0.22
No	438 (85.0)	92 (21.0)	
Ever had sex			
Yes	389 (75.5)	87 (22.4)	(0.1663) 0.68
No	126 (24.5)	26 (20.6)	
Had early sexual debut (< 15 years)^b^			
Yes	26 (6.7)	5 (19.2)	(0.1576) 0.69
No	363 (93.3)	82 (22.6)	
Age disparate sex in past 6 months (>/= 5 years)^b^			
Yes	58 (14.9)	10 (17.2)	(1.0306) 0.31
No	331 (85.1)	77 (23.3)	
Multiple male partners in past 6 months (> 1)^b^			
Yes	81 (20.8)	25 (30.9)	(**4.2561) 0.04**
No	308 (79.2)	62 (20.1)	
Inconsistent condom use with last one (or two) partners ^b^			
Yes	303 (77.9)	69 (22.7)	(0.1309) 0.72
No	86 (22.1)	18 (20.9)	
Inconsistent use of contraceptives in past 6 months^b^			
Yes	258 (66.3)	56 (21.7)	(0.1920) 0.66
No	131 (33.7)	31 (23.7)	
Languishing in terms of emotional well-being			
Yes	74 (14.4)	15 (20.3)	(0.1410) 0.71
No	441 (85.6)	98 (22.2)	
High risk of depression			
Yes	145 (28.2)	31 (21.4)	(0.0373) 0.85
No	379 (71.8)	82 (22.2)	
High Audit C Score (>/=2)			
Yes	248 (48.2)	52 (21.0)	(0.2650) 0.61
No	267 (51.8)	61 (22.9)	

^a^ NEET Not in education, employment or training; ^b^ Denominator is 389 AGYW who had ever had sex

*SES was divided into relatively low and relatively high based on shared features through cluster analysis

### Survey participants’ reports of experiences at the safe space among participants who had visited a safe space

The 113 survey participants who had visited a Safe Space in the past year reported participating in the following services or activities at the Safe Space: 52.2% had had an HIV test, 41.1% had joined a game or fun activity, 32.7% had participated in a sports activity, 24.8% had received counselling to cope with distress, 21.2% had received services from a mobile clinic, 22.1% had participated in a self-defence class, 21.2% had connected to the internet or Wi-Fi, 19.5% had received help with homework, 17.7% had received help from a social worker, 13.4% had participated in a parenting class, and 27.4% had participated in another activity or service at the Safe Space. Most (72.6%) reported that condoms were available at the Safe Space, 85.0% reported that information about health services for young women was available at the Safe Space, and 89.4% reported that the Safe Space was a comfortable space to be in for young women like them.

### Implementers’ views of acceptability of safe spaces and factors affecting implementation

To decide upon the placement of Safe Spaces, and to establish a reliable service referral directory, implementers had conducted situational analyses and service mapping exercises in each sub-district. The method varied by implementer and included: desktop geographical mapping to ensure that the Safe Space was central to key health and social services, schools and transport routes; workshops and consultations with key community stakeholders to identify “hotspots” of vulnerable AGYW; and workshops with AGYW in the district to gather views on the placement and architecture of the physical space and service package. In the design of the programme, it was intended that physical Safe Space hubs would be situated at a community centre in an area that was accessible (walkable and/or via public transport) to AGYW during safe and appropriate times, and well-equipped to deliver services to beneficiaries.

However despite the pre-implementation situational analyses and mapping, implementers who had successfully managed to establish Safe Spaces, described challenges relating to location of Safe Spaces, including safety and accessibility concerns. Safety issues were cited as a key barrier to accessing Safe Spaces. Implementers highlighted the importance of locating Safe Spaces in areas that had been identified as priority areas, specifically those in which no other organisations operated. However, safety was a key concern in some of these areas.

*Finding a safe enough space in a safe enough area, with access to a hall and office space, that was quite a long process. So, we eventually started just begging churches to let us in. (Western Cape, Implementer)*

Concerns around the safety of Safe Spaces was most notable in the Klipfontein district in Cape Town, Western Cape, where implementers were concerned that their Safe Spaces were not ‘safe’ for AGYW and their staff due to gang violence. Some implementers did not feel that it was safe to ask AGYW to come to the established venues, while others were unable to establish Safe Spaces due to these safety concerns. In these areas, implementers felt that it was particularly important to have strong relationships with local community members who could advise on safety concerns and protocols.

*The issue of shootings in Manenberg and Hanover Park, that has made going to these areas very hard; there are areas that we did not go to at all because of these shootings… There are places where we had relationships with stakeholders, as a result they would tell us not to come, not to enter certain areas at certain times because there was a shooting. (Western Cape, Social worker)*

Some implementers explained that they were considering a strategy of implementing flexible/mobile Safe Spaces in these areas, to enable better access for AGYW.

*If I just reflect on Manenberg as an example, our Safe Space that we identified, is located in one area of Manenberg, and obviously there is opposing gang turf… There are girls from one sector of Manenberg that can’t access our Safe Space because they can’t cross turf and they are known from a different part… so that is why we are looking to change from the established Safe Space to something that is more flexible so that we can actually then go into the different areas and access the young women in the different areas… our Safe Space is next to a shop where most of the gang leaders of the area hang out, and we didn’t know that initially… we had one of our staff members stuck at a traffic light with gangs shooting around her, hiding behind a car… so like lots of trauma! (Western Cape, Implementer)*

Respondents explained that safety in implementing areas impacted on the retention of AGYW beneficiaries, and AGYW’s ability to access Safe Space venues and attend sessions consistently.

*It’s very difficult for us at times to retain them in the programme... Because if you’re starting a session… even if it’s IMPOWER (self-defence), Teen Parenting or Grief (counselling). They will drop out! Reason being at times the areas where they are living it’s very dangerous… even for us if we have to go to the community at times, it’s difficult there’s a shooting, the area is vulnerable, the area you can’t walk in… you would find that our offices are in an area where they cannot go that side because the gangs are that side. (Western Cape, Social worker)*

The appropriate positioning of Safe Spaces, in locations that were easily accessible, convenient, and safe, was described as a key factor in their successful use.

*The Safe Space is very far for most of them, so I usually alternate the place, using a hall nearby, a place where people usually go… the community hall… or a school or the church. (KwaZulu Natal, Implementer)*

*A place where you don’t need money for transport and other things. You just go there because it is in the centre of the location… anybody can access it from around the location… it’s a people-centred approach... Some are remote and are not accessible… take the programme or the service to the people. (Mpumalanga, Social worker)*

Experiences across districts differed, with some Safe Space venues described as accessible and appropriate, whilst in other areas, most notably in rural areas, Safe Space venues had been difficult to reach. In some cases, where Safe Spaces and satellites had been inaccessible to AGYW, or poorly located, implementers hired community venues to improve service uptake. An additional factor impacting the accessibility of Safe Spaces related to transport. Using conveniently located community venues as Safe Spaces avoided complications of having to arrange transport for, or provide transport reimbursement money to, AGYW beneficiaries to get them to Safe Space venues.

*We try to find venues closer to where they reside instead of doing this up and down of transport… we find a venue there so that it can be within walking distance. (Western Cape, Social worker)*

Some implementers felt that barriers to accessibility, and challenges in recruiting AGYW, could be addressed through roaming/mobile satellite Safe Spaces offering services.

*We are actually looking at having Safe Spaces that move, like satellites… so that we actually get more access to girls that we are not currently able to reach in the current spaces. (Western Cape, Implementer)*

Challenges in the implementation of the Safe Spaces component of the intervention highlighted the importance of having dedicated physical spaces/venues. However, there had been delays and difficulties in identifying and setting up Safe Spaces which meant that temporary Safe Spaces had to be created within clinics, schools, colleges or hired venues, which negatively impacted on service provision.

*If we could have had the Safe Spaces operating from day one, it would have been easy because they (AGYW) would know I come here and there is a room for biomedical, a room for psychosocial and a room for activities… there is an open place where we can do our CVs and what not. (Free State, Implementer)*

Some implementers based at health facilities had conducive relationships with clinic staff and were assigned a dedicated room in the clinic to be used as a temporary Safe Space, however most struggled to obtain a dedicated space in clinics to receive AGYW. Implementers reported that clinic staff perceived the presence of AGYW programme staff as an added burden, and that they were less accommodating of implementers of programme components other than biomedical components, questioning their contribution to the clinic.

*We seem to not be in clinics as often because they question why are community organizations actually working within a clinic structure… we have gone via the biomedical teams at points because they get access… then they’d know that we are affiliated and then we sort of had more of a right to be in the space because we’re with a biomedical organization or team. (Western Cape, Implementer)*

Implementers who were working within schools but did not have Safe Spaces established, lamented that they could not offer AGYW more comprehensive services, for example access to computers and WIFI, homework support, and assistance to AGYW applying for scholarships and to universities.

*We are based in schools and based out of schools… we don’t have that space where a person can stay for an hour or even two with you assisting them and guiding them and helping them to apply for the learnerships and internships, especially those who have finished matric. (Free State, Implementer)*

Some implementers operating at schools and on college campuses described challenges in cases where they were not provided with a dedicated room or office space, that was appropriately private and confidential, where facilitators could base themselves. Lacking a dedicated space acted as a barrier to access, as AGYW would not know where to come to access services, or did not like being seen with programme staff at school due to fear of being stigmatised by their peers.

*(We need) a space where the facilitator can be found in case a young girl needs something, they will know where to go and find her. Instead of looking for me amongst the teachers, you see. Because others might be afraid to come to me because she is afraid to come to the staffroom where there are all the teachers. (KwaZulu Natal, Implementer)*

AGYW’s concerns over the confidentiality of Safe Spaces emerged as a critical issue. One implementer described confidentiality concerns that emerged when temporary Safe Spaces were set up in schools.

*When I go meet with her at school I will not divulge the reason for my visit, I will talk to the principal or teacher and inform them that there is a case of a learner that I’m attending but the fact that a child will be seen with me at school or leaving school with me is one of the things that make them uncomfortable sometimes. (Free State, Social worker)*

In the design of the intervention, the intention was to leverage on local infrastructure, such as existing governmental multi-purpose community care centres serving youth and vulnerable children and community halls, to act as Safe Spaces where AGYW could ‘drop in’ to access resources and services. However, implementers reported challenges setting up Safe Spaces in community venues due to bureaucracy and political dynamics of working with local power structures, such as ward councillors, traditional leaders, and municipal staff members, who acted as gatekeepers to community venues.

*Most open spaces and halls are taken up by other programmes… communities are quite programme heavy. (Western Cape, Implementer)*

*We struggle for space… you have to ask, especially with things involving councillors... You are told you have to speak to so and so, in order for you to access the area… even though you have spoken about the matter you will find that the issue of getting a space becomes difficult. (Western Cape, Social worker)*

Implementers explained that local Ward Councillors could either enable or constrain their ability to locate and use suitable community venues. Relationships with Ward Councillors and traditional leaders also impacted on community acceptability of the programme and the recruitment of AGYW into Safe Spaces; in some cases assistance was provided by these community stakeholders with recruiting AGYW into Safe Spaces and other programme activities. Fostering good relationships with local leadership and authorities also enhanced the safety of the implementing team in the field, especially in districts where safety was a concern. In some communities, implementers experienced resistance from traditional leaders towards the programme, as it was perceived to be a threat to traditional customs and cultural norms. This was particularly the case in rural areas where the institution of traditional leadership has a stronger presence.

*There are cultural factors because we are staying in a rural area and under the rulership of a traditional council. So there are girls that are still going for virginity testing. Some leaders, like the traditional headmen, are against our programme because they feel like we are introducing the virgin girls into sex… If I’m being honest, I don’t think they will accept topics that deal with sex. Because it is against their culture, their beliefs and they think it is Western culture. (Mpumalanga, Implementer)*

Political interference could undermine not only the Safe Spaces but all aspects of the intervention, for example when Ward Councillors threatened to prevent programme activities in their ward, unless they agree to give financial rewards or preferential access to job opportunities for their kin and political constituents.

*The ward councillors and the municipality, they said that for any programme or activity that will take place we should first start with them, yes. So, they just wanted to be the first priority in the programmes, before the target group. (Free State, Health worker)*

*When we started, we had to sit in one of the constituency offices of the [political party name] somewhere in a hall whereby we were asked questions: Who are you? How many millions are you bringing? … Because there you will find a ward councillor whose interest will be to benefit from the programme. So, one ward councillor would say: ‘you hire my wife or create a good position for my wife, otherwise, you won’t implement in my ward’ … It's all about politics. Now we are going to elections, they look at organisations like us to say, how many people are you going to employ in my ward? (Mpumalanga, Implementer)*

Opinions were mixed amongst implementers as to the best way to engage community gatekeepers to facilitate the establishment of Safe Spaces and recruitment of AGYW. Some implementers motivated for a top-down approach, accessing communities through local gatekeepers like ward councillors and traditional leaders.

*One challenge that we've experienced... the interference of… political and your civil society… I wish we implemented differently… (like in the) PEPFAR approach, you work directly with the stakeholders. You don't start from the ground, because here for you to implement, you have to go to the ward councillor. (Mpumalanga, Implementer)*

Others emphasised the importance of reaching beneficiaries directly and avoiding political interference in the programme.

*The mistake with an intervention is that, if you don’t include the leadership of that community. They will also make sure that your programme is rejected… I usually say it should be an up down process. The district should introduce the programme to the locals and the locals should introduce it to the people on the ground. (KwaZulu Natal, Implementer)*

Contrary to the one of the key objectives in setting up spaces being the provision of biomedical services, implementer respondents explained that AGYW tended to value Safe Spaces for other reasons, such as for accessing the internet for job searching, receiving career guidance, mentorship and assistance with homework and applications for tertiary education from staff, receiving food, toiletries and menstrual management products, as a space to interact with peers, and for general psychosocial support. Having social workers at the Safe Spaces, increased AGYW access psychosocial support, as social workers were able to offer support in private, confidential, and youth friendly venues. Implementers emphasised that especially for AGYW from particularly poor households, being able to come to the Safe Space and access the internet and receive help from peer group trainers to complete homework, had a positive impact on school performance. Offering assistance with homework was also a means to potentially attract AGYW into other programme services including the SRH services; especially for Grade 11 and 12 learners who were very busy with the school curriculum and otherwise difficult to access.

*If you are in school and you do not have internet at home… even if they are crowded at home, these young girls can come to the Safe Space and do their homework and things like that. So, this girl has a safety net… she knows “I have people that can assist me”. (KwaZulu Natal, Implementer)*

Implementers emphasized the importance of proper resource allocation to Safe Spaces in order to ensure their attractiveness to AGYW. Some respondents felt that the resources allocated to Safe Spaces were not sufficient, resulting in Safe Spaces being under resourced, and lacking facilities such as computers and WiFi. Respondents also felt that additional funding to provide AGYW beneficiaries with food, stationary, toiletries and menstrual management products at Safe Spaces would have been beneficial; some field staff even spent their own money to provide these to AGYW at Safe Spaces.

*When it comes to sessions, HTS (HIV Testing) services, individual counselling we are able to render those services. But a child that is in need, who will say “I need stationery, food”, then it stops there, we cannot help any further. We as the field workers decided that to make sure that we work well with the community and make them to trust us again, that each of us who is capable should buy stuff like Vaseline, pads, toothpaste and everything that you can then we put them in a box at our Safe Space. (Free State, Implementer)*

### AGYW’s experiences and acceptability of safe spaces

In general, AGYW beneficiaries shared positive views of the Safe Spaces. Key perceived benefits included access to computers, a safe and quiet environment conducive to studying, access to homework support, assistance with applications, and a space in which to connect with peers.

*They helped us when we got there… typing documents… assisting with their machine and laptops and everything and give us information… We were able to go there and study because it was a safe environment. It was quiet… it was a good place for study. And at that time I was supposed to apply for varsity bursaries, again they are ones who helped me. I just went there and gave them my documents, reports, and everything for the application. They did everything for me. (Mpumalanga, AGYW 15-19 years)*

One perceived value of Safe Spaces among AGYW respondents was that they offered a safe and comfortable place to relax, study, hang out with peers, and access information and support. In particular, AGYW valued access to computers and the internet.

*We just go there (to the Safe Space)... most of the time I just sit down and chill. Sometimes I go with my books, my novels. I just read or study… If I find others I just go there and talk about what affect us as women at this age… we find solutions. (Mpumalanga, AGYW 15-19 years)*

Additionally, AGYW highly valued Safe Spaces as a place to interact with other AGYW, and get peer support.

*We were able to share ideas, advise each other and when I have a problem, I would talk to them and they will help me. It was better than making a decision alone on my own when I have a problem… We were a mixture of girls with different ages, some were 24 years of age and I was 18 years… there was a Safe Space and I felt comfortable when I was there… I was very comfortable when going there… I was never afraid to talk. (KwaZulu Natal, AGYW 15-19 years)*

However, accessing Safe Spaces was not easy for all AGYW beneficiaries. For some AGYW, as mentioned above the location of Safe Spaces, safety of the area, or lack of transport, impeded access.

*They (implementers) sent us a message… and requested that we must come and get our services, but the problem was the issue of distance… so I am unable to go there… to get services (KwaZulu Natal, AGYW 20-24 years)*

For others, competing priorities, such as childcare responsibilities, reduced their ability to access and make use of the spaces.

*What affected my participation was my daughter... I am at home and I am a single parent. I have to take care of her. (Western Cape, AGYW 20-24 years)*

### Safe spaces in the context of COVID-19 lockdowns

In 2020, with the COVID-19 pandemic and the first announcement of a 21-day lockdown with regulations restricting the gathering of people, there was uncertainty among implementers about how the Safe Space model could be maintained. However, most implementers were registered as providers of essential services and they could restart operations soon after the initial lockdown, with the proviso that there was 1.5 m spacing between people in the Safe Spaces. In response to COVID-19 school closures, some implementers continued to run activities with school-based AGYW in Safe Spaces. AGYW respondents described the benefits of Safe Spaces continuing to function during school closures, as some beneficiaries were able to make use of Safe Spaces to study, and get academic support and assistance with homework from the programme staff.

*It was not normal that you study at home through WhatsApp and you don’t get to see teachers when they are explaining. Because for some of us… if you want to understand something a teacher must be in front for you to understand. But, for me it was not that hard because the programme was there for me at the Safe Space. If there were homework and assignments, I could go there (to Safe Space) get help and everything. (Mpumalanga, AGYW 15-19 years)*

These benefits were of additional value during the COVID-19 lockdowns and school closures.

*I was doing my grade 12 last year… during lock down, as you know data and airtime is expensive and by then I was not working… the situation was bad. My two friends and I went there (Safe Space) to ask for assistance… We wanted to study but were unable to go to school… So we were able to go there (Safe Space) and study. (Mpumalanga, AGYW 15-19 years)*

An additional benefit during school closures was that AGYW were able to access Safe Spaces while schools were closed, and have a place to study, get academic support and assistance with homework from the programme staff.

*The programme was there for me at the Safe Space. If there were homework and assignments, I could go there get help. (Mpumalanga, AGYW 15-19 years)*

## Discussion

At the time of our study, well into the second year of the grant period, approximately a quarter of beneficiaries had had accessed any one of the Safe Spaces set up as part of the programme. A large proportion of beneficiaries had not accessed these Spaces and this varied significantly by district, which might represent the variation in district level implementer strategies to set up Safe Spaces, and the varying levels of difficulties they faced. Implementers described challenges identifying and getting permission to use physical spaces, and the community mapping process they undertook at the start of the study did not always overcome the challenges. There was limited availability of physical venues and it was sometimes difficult to work with gatekeepers such as ward councillors and traditional leaders to get permission to access physical venues. The role that ward councillors and traditional leaders play as gatekeepers is a common challenge in development programmes in South Africa [28, 29]. An important dynamic that Safe Space intervention implementers may need to navigate is the friction between traditional leaders and ward councillors, linked to confusion and conflict over the roles of traditional governance, as opposed to political governance systems, in a democratic South Africa. The South African Constitution, although founded on principals of democracy and equality, recognizes the role of traditional leadership especially at local level on matters affecting local communities [30]. This has led to contestations over the role and authority of traditional versus political leaders, including in relation to rights to land and other resources. These tensions may be more pronounced in certain districts, especially in rural areas. If not carefully navigated, this could create a rift in communities if implementing organizations are perceived to align strongly with certain political or traditional figures. Implementers may need to work together to develop guidance on how to navigate these challenges, and to discuss the relative benefits of a top-down approach, whereby they access communities through local gatekeepers, versus reaching beneficiaries directly and avoiding potential political interference in the programme. For example, participatory action research tools could be used at the start of the intervention to understand how the access to physical spaces is negotiated in a community, and perhaps to identify neutral spaces that are not controlled by political or traditional leaders. This could give community beneficiaries the agency to participate in decisions about the selection of physical spaces for the Safe Space intervention [31].

Contrary to one of the key objectives in setting up Safe Spaces for the provision of biomedical services, Implementers believed that AGYW tended to value Safe Spaces for other reasons, especially for the provision of the structural service aspects of the programme. They narrated that AGYW were attracted to Safe Spaces not because of the availability of SRH services, but rather because of the availability of activities and resources to support them in their educational and career goals as well as by recreational activities**.** Implementers believed that Safe Spaces should be designed and equipped in response to AGYW’s interests and needs. The qualitative interviews with AGYW programme beneficiaries provided evidence to support implementers’ perception. AGYW beneficiaries narrated that, in some cases, they were able to continue accessing Safe Spaces, even during COVID lockdowns, where they could study and receive academic support. Unemployment, poverty and low educational attainment are structural drivers of HIV, and Safe Spaces that provide socio-economic interventions focussing on social protection can mitigate AGYW’s HIV risk and vulnerability, and improve AGYW well-being and development [7, 9]. There is evidence that spending time in community spaces and community groups that include an element of adult supervision is protective against HIV incidence and risk [32]. Interventions to strengthen peer networks, and provide curriculum-based education on SRH and gender can improve self-esteem and social networks, as well as improve SRH knowledge and promote safer sexual decision making [33]. Mentorship, together with the provision of safe spaces, has been conceptualized as a key delivery mechanism in HIV programmes in East and Southern Africa that focus on social protection interventions for young women vulnerable to HIV [7]. These findings show that Safe Spaces, in addition to promoting AGYW’s health and providing health services for them, can create an environment that supports their broader well-being.

When implementers secured dedicated physical spaces in which to establish Safe Spaces, they did not always have the funding for the necessary infrastructure and equipment to ensure that they were fully-functional, (including having the resources to provide the socio-economic interventions mentioned above), safe, accessible and attractive to and comfortable for AGYW, with, for example internet, printing facilities, food preparation equipment, and security. Implementers who made use of part-time facilities for their Safe Space hubs or satellites were limited in their ability to resource them fully, and get them fully operational, and only a limited number of services could be offered on a part-time basis. If a Safe Space was not adequately resourced to be able to provide socio-economic interventions, implementers believed their ability to attract AGYW was compromised. Our findings highlight the importance of the provision of socio-economic interventions in Safe Spaces, and this implies that the required resources, staffing complement and facilities are included in the budgets of AGYW programme funding proposals. Adequate consideration of space infrastructure is especially important in the context of the COVID-19 pandemic, to accommodate physical distancing of beneficiaries. One way in which implementers felt that Safe Spaces could be more responsive to socio-economic needs of AGYW, and therefore more attractive to AGYW, was in providing for basic needs, such as food, toiletries and menstrual management products. The provision of food could be a key demand creation feature that drives programme acceptability, community buy-in and the genuine consideration of a holistic programme that is responsive to needs in communities characterised by food insecurity, particularly in the context of the COVID-19 pandemic. The provision of food can also be harnessed as a health promotion tool, through which to educate AGYW about nutrition and equip them with life skills. An important consideration for future combination HIV prevention programmes that include broad-spectrum and socio-economic interventions is whether they can feasibly be implemented within the available budget and resource constraints, without sacrificing fidelity.

Our findings bolster prior evidence showing that AGYW value and benefit from being able to access facilitated social support networks provided in venues such as Safe Spaces, in which they are able to connect with peers, and seek advice and support from trained facilitators [34]. Providing AGYW with the opportunity to build supportive social networks with peers and mentors in the Safe Space of community-based girl groups can build their social, cognitive, economic and health assets [35]. For example, Safe Spaces can build social capital and have been shown to increase agency among adolescent girls and young women [36]. For marginalised and vulnerable youth in society, Safe Spaces can offer temporary respite from experiences of hostility, violence, fear, and danger [37]. Safe Spaces that are co-created and co-produced by adolescents and young people through situated practices are dynamic, emerging through social and peer interactions, offering young people a space in which to to gain a sense of community and connection, free spaces of solidarity, validation, and belonging [37]. In Cape Town, Safe Spaces for men who have sex with men have also been perceived as important to build social capital in this population, and as a long-term strategy for inclusion and emancipation [38]. However, for Safe Spaces to be truly inclusive, power asymmetries within these spaces, and in access to them, need to be considered; ideally, Safe Spaces should enable the inclusion and participation of the most marginalised people [39]. In the case of the AGYW intervention, this would be ensuring access for those AGYW from lower SES strata.

We have shown that even though AGYW might not initially be attracted to Safe Spaces based on the availability of SRH services, if they visit a Safe Space, a large proportion are likely to take up the SRH services on offer. This implies that Safe Spaces that attract AGYW have the capacity to increase the coverage of these services and decrease unmet need in this population. This is important, because AGYW often do not feel comfortable seeking SRH services from health facilities, where they fear being badly treated by health workers, and this is especially the case for younger AGYW. Safe Spaces provide opportunities for young people to practise and learn to discuss and access SRH services and products within a protected environment and to gain confidence. Of concern, the results of our survey show that beneficiaries who were classified as in the relatively lower SES category were significantly less likely to have accessed and spent time at a Safe Space than those classified as higher SES. This finding was reinforced by the qualitative study findings which describe how some of the Safe Spaces were too far away and AGYW would need money for transport to access them. This highlights the importance of strategies to ensure the accessibility and acceptability of Safe Spaces for the most vulnerable AGYW. These could include co-designing of Safe Spaces with the groups who are currently under-served, covering transport fees, outreach activities to encourage participation, exploring barriers to access, and roaming/mobile Safe Spaces.

We found that AGYW were attracted to Safe Spaces to relax with peers, “play”, and participate in cultural activities. This is important because restrictive gender norms often undermine adolescent girls ability to participate in community life, sports and games [40]. In recognition of this, it is common for sport to be incorporated into HIV programming for AGYW programmes, including the Safe Space interventions in South Africa. Such recreational activities offer AGYW opportunities to play within a protective environment and can create a safe space for discussion and learning, and encourage them to advocate for their rights [41]. Sports participation has been conceptualized as a developmental resource for adolescents in ways that influence sexual behaviour and reduce HIV risk [40], and there is evidence that sport-based HIV prevention is a promising approach [41, 42], enabling adolescents and young people to develop their capacities for agency, self-regulation and self-protection. The incorporation of play, rest and recreation within Safe Spaces should be conceived of as critical enablers of improved health and wellbeing, recognising young people’s holistic journey to adulthood. At a policy level, it is important to recognize *Article 12 of the African Charter on the Rights and Welfare of the Child* as complementary to the South African SRH policies, including the *2012 Integrated School Health Policy*, and *2017 Department of Basic Education National Policy on HIV, STI’s and TB for Learners, Educators, School Support and Officials in all Primary and Secondary Schools in the Basic Education Sector*. By integrating these guidelines into a coherent conceptual framework, we can acknowledge and affirm that children, adolescents and young people pursue pleasure, self-directed activity, and exploration as part of their development, and that to programme effectively we need to recognise and respect this. Socio-economic status and the physical environment in which adolescents and young people live enables or constrains their capacity to adopt good nutritional habits and practice play/sport/exercise [43]. The lack of safe spaces has been identified as a key barrier to physical activity practices, amongst young people in sub-Saharan Africa (Jesson et al., 2020). Research amongst young people in resource constrained communities in sub-Saharan Africa has highlighted the need for youth-friendly safe spaces not only to practice sport but also to socialise in a safe environment, which would also serve to create an enabling environment for young people’s personal development and empowerment [43].

Implementers noted the difficulties of implementing AGYW programme activities in school settings because these settings did not have extra offices or space for dedicated health programmes. Establishing Safe Spaces at schools was not part of the proposed AGYW programme and budget. Our findings suggest that school-based Safe Spaces would facilitate the implementation of AGYW programme activities, and would also increase participation and acceptability and reduce transport costs. School-based Safe Spaces have the potential to create zones of ‘autonomy’ within schools which might otherwise be resistant to making SRH services available in school premises. However, many schools will not have the facilities and structures to accommodate Safe Spaces. If school-based Safe Spaces are part of future programmes, the budgets and plans need to include the necessary architectural changes to the school built environment.

In terms of the ‘appropriateness’ of the Safe Spaces component for context of the communities in which the intervention is being implemented, key issues that emerged in the findings related to challenges in offering services to AGYW at Safe Spaces during safe and appropriate times, and challenges with ensuring the appropriate positioning of Safe Spaces, in locations that were easily accessible, convenient, and safe. Additionally, although in this programme the Safe Spaces were not school-based, a challenge with school-based Safe Spaces would be difficulty in securing dedicated physical spaces that are appropriately private and confidential for service provision.

### Limitations

Safe Spaces established as part of the AGYW programme were not deliberately branded, and therefore it is not possible to know whether AGYW participants’ understanding of Safe Spaces was aligned to the venues that implementers conceived of as Safe Spaces. However, the activities and resources participants described at the Safe Spaces they reported attending, were aligned to those provided at the AGYW programme Safe Spaces, which supports the validity of the survey measures. The Safe Space intervention was not implemented at a uniform pace across districts and implementers, and there was no requirement for implementers to adhere to a time-bound standard roll-out plan. We were not able to explore the district-level factors associated with access to Safe Spaces due to the small district sample size. A limitation of conducting the study among beneficiaries who were enrolled in the early period of the AGYW programme, which was also the period in which the COVID-19 pandemic occurred, is that the findings do not reflect the full potential of the intervention when all Safe Spaces would have been effectively implemented. A further limitation was that the success of the sampling strategy was dependent on beneficiaries being contactable by the implementers and the researchers, predominantly by phone. Those who were not contactable by phone are likely to be different to, and possibly more vulnerable than those who were contactable, and this may have introduced a bias in the study findings, in that we may not have captured the experiences of the most vulnerable AGYW. The study population, registered beneficiaries of the AGYW programme, implies that our findings are not necessarily representative of all AGYW in the intervention communities, many of whom would not have been beneficiaries. We did not include measures of intimate partner violence (IPV) and sexual violence in our survey, and therefore we “fear of partner” to indicate risk of IPV. The validity of this measure as an indicator of vulnerability to IPV and sexual violence is unknown. Our study demonstrates the potential of Safe Spaces to increase coverage of HIV prevention interventions, but further research is needed to investigate whether Safe Spaces are indeed effective interventions to increase the coverage of such interventions.

## Conclusions

When the Safe Spaces established as part of a South African combination HIV prevention programme were resourced to promote AGYW’s socio-economic goals (such as employment and educational progress) and to meet AGYW’s needs for social interaction and peer engagement, they were acceptable to, and popular among the AGYW programme beneficiaries. The popularity of Safe Spaces offering such resources can be harnessed as an entry point for engaging AGYW in biomedical HIV prevention or treatment services or in SRH care. Our study found that a large proportion of AGYW who visited the Safe Spaces used the SRH services on offer, and this demonstrates the potential of Safe Space interventions to reduce the unmet need for SRH care in this population. Poverty and poor educational attainment are underlying causes of AGYW’s vulnerability to HIV and adverse SRH outcomes [44–46]. Safe Spaces with structural interventions such as those supporting AGYW’s employment and educational progress address the underlying causes of AGYW’s vulnerability to HIV, and they also create an enabling environment for the synergistic preventive action of biomedical and behavioural interventions (https://www.paho.org/en/topics/combination-hiv-prevention).

We found that approximately half-way through the three-year grant period, one quarter beneficiaries of the AGYW programme had accessed a Safe Space, indicating a relatively high coverage and the potential to increase the reach in the remainder of the grant period. We have shown that the Safe Space intervention in this South African combination HIV prevention programme was less accessible to the poorest AGYW and this highlights the importance of deliberatively designing Safe Space interventions to be accessible for the most vulnerable. As Safe Space interventions are rolled-out, it is important to monitor disparities in access to them by SES. SES inequalities are associated with inequities in SRH among adolescents, for example, poorer adolescent girls (compared with wealthier) face more barriers in meeting their SRH needs [47]. Our study draws attention to the potential value of school-based Safe Spaces, and these may be one approach improving access among the poorest young adolescent girls. Other approaches might include mobile safe spaces to improve access in remote areas.

Our study has demonstrated the political, structural, and financial challenges of implementing Safe Space interventions. Participatory action research at the start of an intervention might be one approach to navigate the political challenges of identifying physical spaces in which to set up Safe Spaces [31]. A cost analysis should guide decisions about the resources required for establishing or possibly building the physical spaces, and for providing the structural, behavioural and biomedical interventions, and should inform future budgets for combination HIV prevention intervention delivery though Safe Spaces. Additional costing considerations may need to be made in the context of pandemics, such as COVID-19. For example, in addition to physical spaces, online platforms at no cost to AGYW might be required to deliver psycho-social support interventions.

## Data Availability

The qualitative datasets generated and analysed during the current study are not publicly available due to the potential to violate the privacy of the human subjects, but are available from the corresponding author on reasonable request. The quantitative is not yet publicly available because the authors are in the process of seeking permission from the funders to make it publicly available, but the dataset is available from the corresponding author on reasonable request.

## Footnotes

1 For further details about recruitment, see project report (https://www.samrc.ac.za/sites/default/files/attachments/2021-07-27/HERStory2_Qualitative_Process_Evalutaion.pdf)
